# AFAL: a web service for profiling amino acids surrounding ligands in proteins

**DOI:** 10.1007/s10822-014-9783-6

**Published:** 2014-08-02

**Authors:** Mauricio Arenas-Salinas, Samuel Ortega-Salazar, Fernando Gonzales-Nilo, Ehmke Pohl, David S. Holmes, Raquel Quatrini

**Affiliations:** 1 Centro de Bioinformática y Simulación Molecular, Facultad de Ingenieria, Universidad de Talca, 3465548 Talca, Chile; 2 Facultad de Ciencias Biologicas, Universidad Andres Bello, Santiago, 8370146 Santiago Chile; 3 Department of Chemistry & School of Biological and Biomedical Sciences, Biophysical Sciences Institute, Durham University, South Road, DH1 3LE Durham, UK; 4 Fundacion Ciencia & Vida, Santiago, 7780272 Nunoa Chile

**Keywords:** AFAL, Protein–ligand interactions, PDB, Drug design, Enzyme engineering, Bioinformatics

## Abstract

With advancements in crystallographic technology and the increasing wealth of information populating structural databases, there is an increasing need for prediction tools based on spatial information that will support the characterization of proteins and protein–ligand interactions. Herein, a new web service is presented termed amino acid frequency around ligand (AFAL) for determining amino acids type and frequencies surrounding ligands within proteins deposited in the Protein Data Bank and for assessing the atoms and atom-ligand distances involved in each interaction (availability: http://structuralbio.utalca.cl/AFAL/index.html). AFAL allows the user to define a wide variety of filtering criteria (protein family, source organism, resolution, sequence redundancy and distance) in order to uncover trends and evolutionary differences in amino acid preferences that define interactions with particular ligands. Results obtained from AFAL provide valuable statistical information about amino acids that may be responsible for establishing particular ligand–protein interactions. The analysis will enable investigators to compare ligand-binding sites of different proteins and to uncover general as well as specific interaction patterns from existing data. Such patterns can be used subsequently to predict ligand binding in proteins that currently have no structural information and to refine the interpretation of existing protein models. The application of AFAL is illustrated by the analysis of proteins interacting with adenosine-5′-triphosphate.

**Electronic supplementary material:**

The online version of this article (doi:10.1007/s10822-014-9783-6) contains supplementary material, which is available to authorized users.

## Introduction

Many proteins require small molecular ligands or cofactors in order to fulfill their specific biological roles. These ligands include a large number of small organic biomolecules, such as adenosine-5′-triphosphate (ATP) or heme as well as inorganic ions and molecules, for example transition metal ions including Fe^2+/3+^ Cu^+/2+^ or Zn^2+^ [1]. Such ligands can be loosely or tightly bound to the protein and participate directly or indirectly in catalysis. Protein ligand interactions are highly diverse with respect to fold and coordination environment [2]. A wide variety of chemical groups, including carboxyl, imidazol, indol, thiol, thioeter, hydroxil moeties, etc., participate in the coordination of diverse ligands through different amino acid residues and motifs. Understanding the structural and dynamical aspects of their binding is essential for the overall comprehension of the structure and function of proteins.

The study of the specific interaction of a protein with its ligand is an active research field because of the implications this has in the overall understanding of the structure and function of proteins, and in particular in the fast-growing area of structure based drug design [3]. A number of free applications, tools and services have been posted on the web [4, 5] that aim to predict and characterize protein–ligand interaction through their binding affinity and energetics. Sophisticated tools like 3D structure–activity relationships (3D QSAR) link experimental and theoretical data to predict such interactions [6, 7]. Furthermore, molecular simulation and docking [8] and molecular interaction fields [9] have also proven very useful in the area of structure-based drug design.

In today’s research environment, a wealth of experimental and theoretical structural data is available. There are currently 96,980 macromolecular structures stored in the RSCB Protein Data Bank (PDB, January 2014), 70,908 of which correspond to proteins that contain ligands (small molecules) as part of their structures and which belong to diverse organisms including *Escherichia coli* (17.4 %), *Thermus thermophilus* (18.3 %), *Haloarcula marismortui* (10.9 %), *Saccharomyces cerevisiae* (8.4 %), *Bos taurus* (9.7 %), *Homo sapiens* (9.1 %), and others [10–12].

Ligands in the PDB currently encompass 16,447 different chemical components, ranging from single atoms (e.g. Na^+^) to complex pyrrolic rings (e.g. heme) and non-standard polymers [10, 13]. This makes the information stored in the PDB a very important source for data mining and analysis.

Other web accessible resources such as SuperLigands [14], Ligand Expo [15] and the IMB Jena image library of biological macromolecules [16] retrieve additional information on small molecules found in the PDB and help to identify ligands that are likely to bind a given protein structure. However, neither prediction nor interpretation of these interactions is straightforward. In the absence of additional resources for the retrieval of spatial information, this massive amount of highly sophisticated data simply represents a catalogue of the interactions of individual proteins with individual ligands, and does not contribute directly to an understanding of protein and ligand functions nor to the underlying rules that govern such interactions.

Several studies have been carried out that analyze amino acid preferences at ligand binding sites [17, 18]. General trends have emerged from these studies, such as an enrichment of Gly, Ser, Arg and Tyr in binding sites that correlate to the role of these amino acids in secondary and tertiary structure formation [16]. Similarities in the amino acid environment at certain binding site has also been evaluated from an evolutionary perspective [19, 20]. Comprehensive analysis of well-defined structural motifs of ligand-binding sites has revealed that most structural motifs are confined within single protein families or superfamilies and are associated with particular ligands [21]. No method applied so far to the exhaustive all-against-all comparison of ligand-binding sites found in PDB has been effective in deriving insights into the nature of the interactions, based possibly on structural (fold) as well as evolutionary (phylogenetic) constrains. Therefore, alternative tools for the analysis of the interactions between proteins and their ligands across protein families and phylogenetic backgrounds are required.

By integrating conventional data mining techniques with structural biology analysis tools the amino acid frequency around ligand (AFAL) application analyzes the protein structures stored in PDB and identifies the amino acids and atoms involved in the interaction with any ligand (e.g. drug molecules, co-factors, etc.). AFAL displays the protein–ligand interaction atomic distances and calculates the frequency of the amino acids that surround a particular ligand and the frequency of the atomic interactions per residue. Identification of the most likely pattern of residues implicated in the binding of given ligand, independently of fold and phylogenetic background, can be useful not only to derive insights into the nature and evolution of specific protein–ligand interactions and the understanding of molecular and atomic level interaction mechanisms but also in applied studies related to drug design or modification of functional groups in proteins of biotechnological interest.

## Methods

**Fig. 1:**
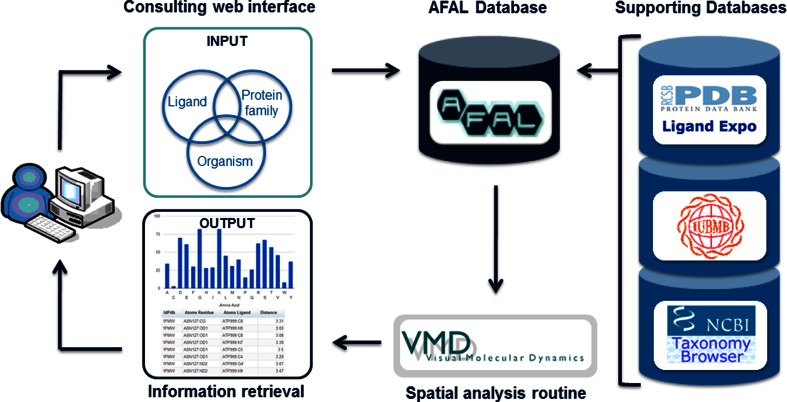
Architecture of the AFAL application. The input is entered by the user through the AFAL consulting web interface. After choosing filters (listed in Fig. 2), AFAL retrieves available structural data in the PDB matching the query that is then analyzed by the VMD software. The results (output) are stored in the AFAL database and sent to the user’s e-mail. If the query was run previously by another user, the stored result is immediately dispatched to the user

AFAL has been compiled using pre-existing and publically available resources and software packages (Fig. 1) such as the PDB database [10–12], its Ligand Expo Search feature [15], the IUBMB Enzyme Nomenclature Database [22], the NCBI Taxonomy Database [23] and the VMD software [24]. The AFAL web service consists of three major components, the AFAL Database, the Consultation web interface and the Spatial analysis routine (Fig. 1), described in detail bellow.

### The AFAL database

A local database was created to facilitate quick access to the structural data stored in PDB and to adequately classify the information to be retrieved in each search according to user selected filters. The database was built using a MySQL engine version 14.14. To populate the database and to classify the PDB files, multiple scripts programmed in Perl language were generated. More than 90,000 files from PDB were accordingly classified into proteins with a ligand, proteins without ligand, type of ligand, protein family, organism and crystal resolution. The local database also stores the results of each new query consulted. This facilitates the access for new users to pre-calculated amino acid frequencies around commonly consulted ligands. The AFAL database is automatically updated every month to include actualized PDB files in every new search.

### Consulting web interface

**Fig. 2:**
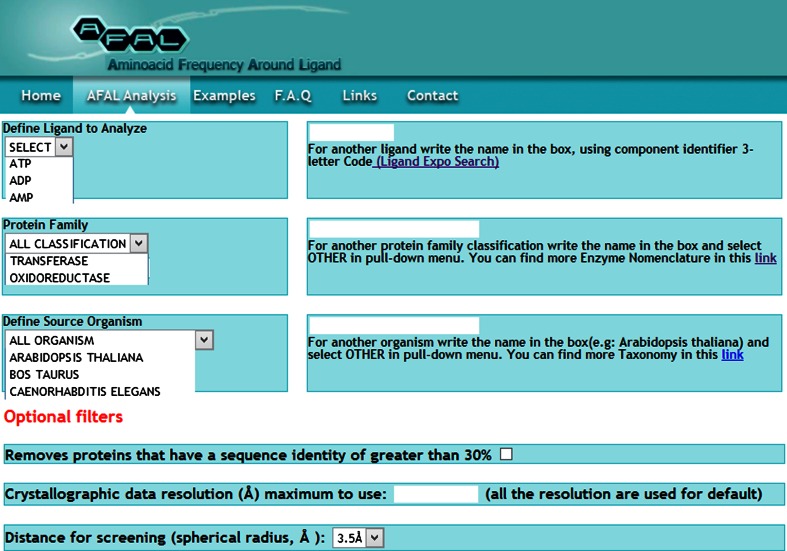
The AFAL application web interface. Various filtering parameters, pre-established menus and hyperlinks for defining the query and retrieving the information are depicted

The web interface, created in html and php, provides the user with a friendly and easy to use form for entering query data and selecting the filtering parameters (Fig. 2). A search is initiated with the selection of the ligand of interest. The user may choose the ligand from a pre-established menu or by entering the ligand name according to the three-letter code used in PDB. To facilitate this step, AFAL redirects the ligand three-letter code search towards the PDB Ligand Expo Feature [15]. Other filtering parameters can be left in their default options at this stage, but the result produced will be very general and all encompassing. The user may then narrow down the search to uncover preferences or tendencies in the usage of certain residues in the coordination of a given ligand using adequate filtering criteria. The options available in this version of AFAL are a protein-family filter and a species-filter, which restrict the analysis to a particular group of proteins (e.g. Kinases) or a particular source organism (e.g. *Homo sapiens*). Both filters have customized menus, using a list of pre-defined protein families or organisms. If the protein family or source organism is not in the list, the user can type in the respective name(s) using the IUBMB Enzyme nomenclature database link and/or the NCBI taxonomy database link. Since many of the structures in the PDB database are highly similar or even identical, a further filter avoids biasing of the results towards multiple counting of interactions by culling protein sequences in PDB by sequence identity using PISCES [25]. The default sequence-identity cut-off to remove highly similar proteins from the data set has been set at 30 % sequence identity. The user may select or deselect this option at choice. In addition, the user may further restrict the search space by selecting the crystallographic resolution of the target proteins and by setting a cut-off value for the protein–ligand screening distance. The default value is set at 3.5 Å, a distance at which both covalent and strong electrostatic interactions occur [26].

### Spatial analysis routine

To identify the amino acids and atoms that surround a given ligand AFAL makes use of the spatial analysis functions of the VMD software [24]. Using a script programmed in Tcl language [27] hundreds or thousands of protein structures (PDB files) that meet the filtering criteria set by the user and selected via the AFAL database can be automatically analyzed. The powerful module of VMD, atom selection method, guides the search of the atoms in the protein under analysis found at a given distance from the ligand of interest within the spherical radius defined by the user. All residues within this radius can be recovered from the PBD fitting the filtering criteria together with the closest atom to the ligand and their interaction distances, trough a drop-down menu and accompanying table.

### Information retrieval

**Fig. 3:**
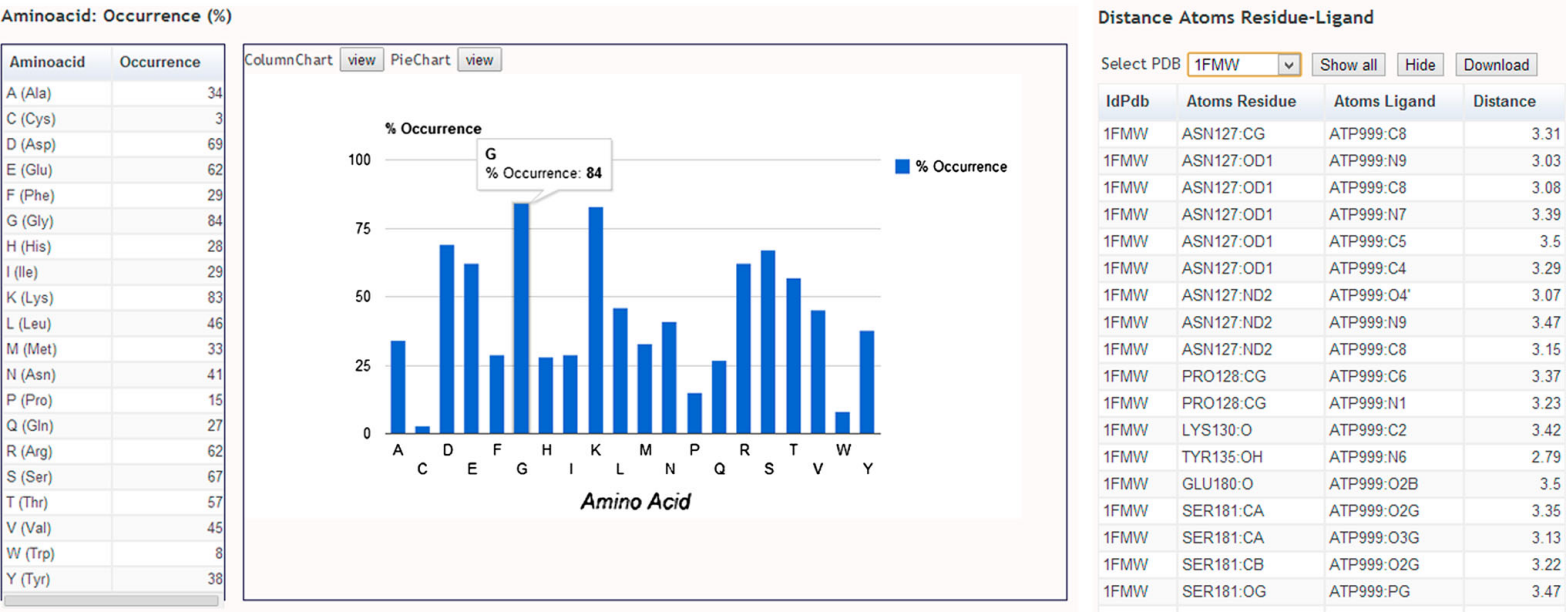
The AFAL results section web interface. The figure displays the result of a standard analysis for the ligand ATP. In the interactive histogram, the frequency of occurrence of each amino acids surrounding the ATP within a 3.5 Å radial distance is calculated with respect to all the ATP-binding structures stored in PDB (default filter option). The *dialog box* shows that the Gly residue is 84 % present in all the PDBs file analyzed that use ATP as ligand. On the right, the details of the interactions of ATP with protein PDBID:1FMW are shown

The AFAL output consists of a frequency table and an accompanying interactive histogram (Fig. 3). All amino acids occurring around a certain ligand, inside of the spherical radius distance set for any given protein structure selected according to filtering criteria defined by the user are scored for presence or absence. Occurrence of a residue is then expressed as a percentage value of the total of PDB structures analyzed in a given interrogation. Results are tabulated in a spreadsheet, graphed and displayed in a web page. The link generated is sent to the user by e-mail within a few minutes. Preexisting calculations stored in the AFAL database are also displayed on screen immediately. Two kinds of tables are produced. The first lists the PDB files selected based on the users filtering criteria and displays the amino acid residues involved in the interaction with the ligand. The second table details the atoms involved in the interaction with the ligand and the interaction distances for all PDB files involved in the analysis (Fig. 3). This information is very useful for the characterization of the interaction microenvironment of any given ligand. This information can be downloaded by the user for further in-house analyses.

### Website

The AFAL service is freely available for noncommercial use at http://structuralbio.utalca.cl/AFAL/index.html. AFAL is supported by Center of Bioinformatics from the University of Talca and will be constantly updated and maintained to ensure reliable operation even when some of the underlying tools are changing.

## Utility and discussion

### ATP-binding proteins as test case

To demonstrate the utility of AFAL to identify amino acids relevant in the coordination of particular ligands, a well characterized protein–ligand interaction was chosen as an example. ATP has essential roles in all forms of life. Characterization of the interaction of this molecule with specific amino acids is of great importance for understanding enzymatic mechanisms and for drug design. The ATP molecule is composed of an adenine base linked to three phosphate groups via a ribose. When bound to proteins, one or more magnesium ions are often found in coordination with the negatively charged phosphate groups. ATP is a multifunctional nucleotide used for many biochemical reactions that require energy via hydrolysis of the γ-phosphoester bond [28] and participates in many cellular processes including cell signaling via phosphorylation of proteins [29], transport through the ABC transporters [30], DNA repair by DNA binding proteins [31] and is the main substrate in signal transduction pathways by kinases [32].

**Fig. 4:**
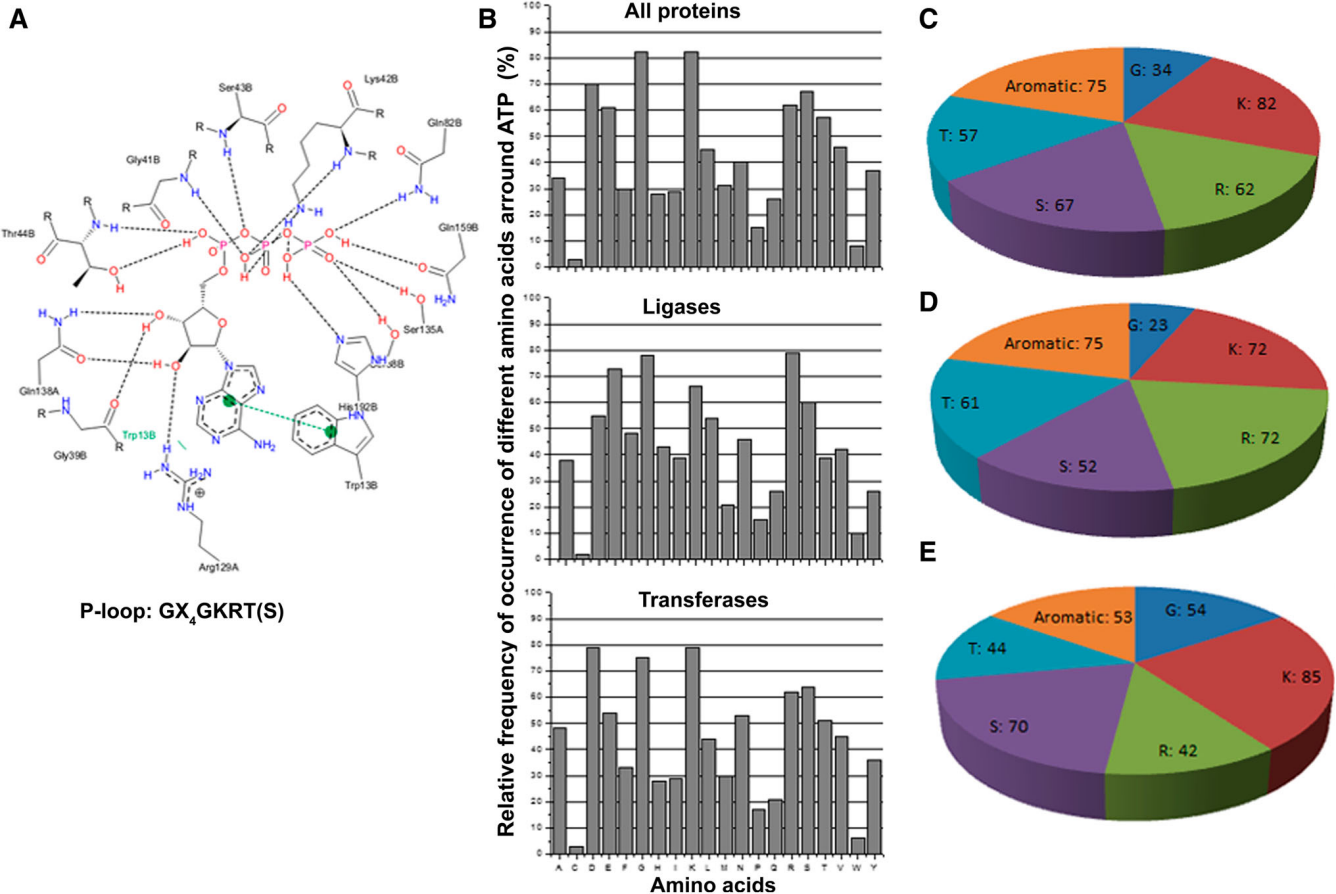
AFAL results for ATP-binding proteins. **a** Walker motif structural representation obtained from PDB entry 2R6G [38] using PoseView software [39]. **b** Relative frequency of occurrence of each amino acid around ATP at 3.5 Å for all protein families baring this ligand and for transferases and ligases only. **c**–**e** Walker motif amino acid residue distribution for all protein families in all organisms available in PDB (**c**) or in *E. coli* (**d**) and *H. sapiens* (**e**) only

Several studies have been carried out to characterize the amino acids involved in recognition of the phosphate groups and the adenine moiety. Recognition of phosphate groups requires the consensus sequence of GXXXXGKT(S), with serine substituting threonine in some cases [33]. This motif is more popularly known as the Walker motif or P-loop [34] (Fig. 4a). In turn, adenine–protein interactions depend on the adenine base capacity to establish hydrogen bonds, π–π stacking interactions and cation–π interactions. Aromatic amino acid Phe, Tyr and Trp are involved in the π–π stacking interactions forming the A-loop (aromatic loop) motif while positively charged residues Lys and Arg are responsible for the cation–π interactions [28, 30].

In the November 2013 PDB release, there were 768 ATP-binding protein entries interacting with different protein families in a range of distances, the most common of which are the Transferases with 268 proteins (enzymes transferring a group, for example, phosphorus-containing groups) and Ligases with 104 proteins (enzymes that catalyze the joining of two molecules with concomitant hydrolysis of the diphosphate bond in ATP or a similar triphosphate) [22]. These ATP-binding proteins use different ways of binding the phosphoryl moieties as well as the adenine base, but the most common sequence and structural motif for binding ATP is the Walker motif. AFAL was used to uncover trends in the amino acid preferences of ATP binding pockets by assessing the relative frequency of occurrence of each amino acid around ATP at 3.5 Å in all or in certain groups of ATP-binding proteins. To ascertain if the trends uncovered by AFAL are meaningful the reader is referred to the reference table and accompanying chart in the frequently asked questions section of the AFAL web page (http://structuralbio.utalca.cl/AFAL/faq.html) showing overall frequencies of occurrence of each amino acid in proteins in general and/or in particular protein families (Table S1).

As shown in Fig. 4b, Gly (84 %), Lys (83 %), Arg (62 %), Ser (67 %) and Thr (57 %) are one the most frequently occurring amino acids identified by the AFAL algorithm that surround ATP in the available ATP-binding proteins from PDB (Fig. 4b) and in different source organisms (Fig. 4c–e). These are typically present in the P-loop motif (Fig. 4a; Table S2). Narrowing down the search to specific protein families (Fig. 4b), additional tendencies in the use of certain residues for the coordination of ATP emerge. It can be concluded that AFAL correctly identifies the three conserved residues (Gly, Thr, Ser) that define the P-loop motif described for the Transferase family and is in agreement with previously described trends for binding site in general [17]. The occurrence of the positively charged residues Arg and Lys, potentially involved in adenine–protein cation–π interactions [28, 35, 36], was also observed (Table S3). The analysis of the proteins belonging to the Ligase family shows a similar tendency, although an increased occurrence of Arg over P-loop motif residues suggests that adenine–protein cation–π interactions are present in this protein family.

Table 1 lists the observed interactions of the phosphates of ATP with amino acid residues of the Walker motif and provides information regarding the frequency of these interactions in the PDB and the average distance of the interactions between the respective amino acid residues and one of the phosphates of the ATP molecule. Gly interacts principally with the β-phosphate of ATP, Lys with the β-phosphate and γ-phosphate, Thr with all three phosphates almost equally but showing a slight preference for the γ-phosphate and Ser with the γ-phosphate. The observed distances of these interactions descends from 3.4 Å for Gly to 3.27 Å for Ser. These findings are in agreement with previous studies [34, 37] and validate the utility of AFAL for analysis of protein–ligand interaction patterns. In addition, these results demonstrate the power of AFAL to find novel amino acid–ligand interactions.

**Table 1:** Identification of amino acid residues from the Walker motif that interact with the α-, β- and γ-phosphate groups of ATP within a distance of 3.4 Å or less using data derived from the PDB. Listed are the number of such interactions and the average distance (Å) between the residues of the Walker motif and the respective phosphate groups of the ATP

Walker motif amino acid residue	ATP phosphate interaction	Number of interactions in PDB	Average distance from residue to phosphate (Å)
Gly	α-Phosphate	28	3.35
	β-Phosphate	158	3.40
	γ-Phosphate	41	3.38
Lys	α-Phosphate	43	3.33
	β-Phosphate	184	3.34
	γ-Phosphate	146	3.30
Thr	α-Phosphate	38	3.35
	β-Phosphate	28	3.40
	γ-Phosphate	47	3.33
Ser	α-Phosphate	20	3.27
	β-Phosphate	44	3.37
	γ-Phosphate	95	3.27

A reference table that summarizes the most common contact types made by the amino acid and the most common functional groups from ligand atoms included for comparative purposes in supplementary Table S3 and is also available for on-site consultation by interested users at the bottom of the AFAL results page.

Additional examples of the use of AFAL are provided in the web site.

## Conclusions

AFAL offers an automated solution for the analysis of interactions between proteins and their ligands across protein families and phylogenetic backgrounds using crystallographic data stored in the PDB database. The results obtained from AFAL provide valuable statistical information about the amino acids, atoms and distances that may be responsible for establishing any particular ligand–protein interaction, helping to compare the ligand-binding sites of different proteins and to uncover general as well as specific interaction patterns from existing data. It is anticipated that AFAL will provide an excellent opportunity to extract valuable information on the evolution of protein–ligand interactions and help suggest functions for unknown proteins containing potential ligand binding sites.

## Electronic supplementary material

Below is the link to the electronic supplementary material.

Supplementary material 1 (XLSX 33 kb)

Supplementary material 2 (XLSX 103 kb)

Supplementary material 3 (XLSX 76 kb)
